# Low frequency ultrasonic dataset for pulse echo object detection in an isotropic homogeneous medium as reference for heterogeneous materials in civil engineering

**DOI:** 10.1016/j.dib.2022.108235

**Published:** 2022-05-01

**Authors:** Stefan Maack, Stefan Küttenbaum, Benjamin Bühling, Ernst Niederleithinger

**Affiliations:** Bundesanstalt für Materialforschung und Prüfung (BAM)^1^, ^2^, Unter den Eichen 87, Berlin 12205, Germany

**Keywords:** Ultrasound, Pulse-echo method, Non-destructive testing, Reconstruction algorithm, Reference material, Validation, Piezoelectric transducer, Civil engineering

## Abstract

The dataset presented contains ultrasonic data recorded in pulse echo mode. The investigated specimen is made of the isotropic homogeneous material polyamide and has a drill hole of constant diameter running parallel to the surface, which was scanned in a point grid using an automatic scanner system. At each measuring position, a pitch-catch measurement was performed using a sampling rate of 2 MHz. The probes used are arrays consisting of a spatially separated receiving and in-phase transmitting unit. The transmitting and receiving sides each consist of 12 point-shaped single probes. These dry-point contact (DPC) probes operate according to the piezoelectric principle at nominal frequencies of 55 kHz (shear waves) and 100 kHz (longitudinal waves), respectively, and do not require a coupling medium. The measurements are performed with longitudinal (100 kHz) and transverse (55 kHz) waves with different geometric orientations of the probe on the measurement surface. The data presented in the article provide a valid source for evaluating reconstruction algorithms for imaging in the low-frequency ultrasound range.

## Specifications Table

**Table:** 

Subject	Engineering
Specific subject area	Ultrasonic data in time domain from an isotropic homogeneous medium for geometrical representations of the inner structure with reconstruction algorithms
Type of data	Time series
How the data were acquired	Automated scanning system: Developed by BAM Probe: M2502 – Dual Aperture Shear Wave DPC Transducer Array (Acoustic Control Systems - ASC Group), M2503 – Dual Aperture Longitudinal Wave DPC Transducer Array (Acoustic Control Systems - ASC Group) Driving signal generator: In-house development by BAM Data Acquisition: USB-6361 (National Instruments Corp.) Software: LabVIEW© (National Instruments Corp.- Scanner control and data acquisition developed by BAM, Germany)
Data format	Raw data
Description of data collection	The specimen has a rectangular cross-section with a constant diameter drill-hole running parallel to the measurement surface. Point-wise ultrasonic pulse-echo measurements are performed on an equidistant grid from the measurement surface using an automated scanning system. The full dataset contains 6161 measurement points. The probe uses spatially separated transmitter and receiver units.
Data source location	Institution: Bundesanstalt für Materialforschung und -prüfung (BAM) City/Town/Region: Berlin Country: Germany
Data accessibility	Repository name: Harvard Dataverse Data indentification number: https://doi.org/10.7910/DVN/KVN7CY
Related research article	S. Maack, S. Küttenbaum, N. Epple, N. Aligholizadeh (2021) Die Ultraschall-Echomethode - von der Messung zur bautechnischen Kenngröße, Beton- und Stahlbetonbau, 1–12. https://doi.org/10.1002/best.202000091

## Value of the Data

• The dataset was recorded on a specimen made of polyamide. Common building materials such as concrete have a highly heterogeneous structure, which leads to strong sound attenuation due to the scattering and absorption of the ultrasonic signal. In addition to the natural heterogeneity of the material, cracks and pores that inevitably occur during the casting of concrete play a significant role in the scattering of ultrasound. Overall, the strong scattering of ultrasound waves means that some objects can only be reliably detected by using reconstruction algorithms. As the algorithms proposed by different authors, institutions or corporations are hard to compare due to the fact that they are usually demonstrated using measurements made with different devices and setups and on different objects, we find it useful to provide a well-documented dataset, which also excludes some of the variabilities in datasets made on concrete. Volume scattering does not occur in an isotropic homogeneous material such as polyamide. The test specimen has geometric dimensions that correspond to the actual size of real components and the resulting testing tasks. Besides the volume waves (longitudinal and transverse), wave types such as surface waves are additionally generated, which can have a disturbing effect during evaluation. The recorded wave field is therefore very realistic in comparison with real measurements. This dataset can therefore be used as a reference for investigating the propagation of ultrasound under realistic boundary conditions without influences by scatter.
• The specimen has a high industrial machining quality and ensures a very high accuracy regarding the geometrical dimensions. This high degree of geometric precision is difficult or almost impossible to achieve when manufacturing test specimens from concrete. Furthermore, the measurement data was gathered using a two-dimensional automated scanner system with a pneumatic device for moving, coupling, and lifting the test probe. This ensures both a very high geometrical precision in data acquisition and a constant contact pressure at each measuring point.
• The characteristic properties of the sound fields in polyamide are given by specifying the directivity patterns of the longitudinal and transverse wave probes used. The geometrical dimensions of the probe and the aperture, respectively, are provided.
• Different stakeholders can benefit from the dataset. It is possible to compare the results of different reconstruction algorithms using the dataset (e.g. in round robin tests). Furthermore, the effects of varying input parameters in a reconstruction calculation can be analyzed.
• Using the experience gathered with the presented dataset, additional test specimens and datasets can be developed and acquired for different testing scenarios.

## 1 Data Description

This dataset [1] contains raw data of ultrasound measurements on a polyamide reference specimen conducted at Bundesanstalt für Materialforschung und -prüfung (BAM), Berlin. The BAM internal specimen identifier is “Pk218“ (German: Probekörper 218, abbr.: “Pk218“). The measurements were conducted using the pulse-echo method, where transmitting and receiving transducers are placed on the same face of the specimen. The upper surface of the specimen was defined as measuring area (X-Y-plane). The aim of the measurements was to determine both the specimen thickness and the position of a hole drilled parallelly to the measuring area.

The dataset contains the raw data and geometrical (lateral) information of the ultrasonic measurements. These are stored in two data formats: Python format (*.npy, Python version 3.8) and a Comma-Separated Value format (*.csv) based on Shafranovich [2].

In Python format, the data is stored in a three-dimensional array. Each dimension in the 3D-array corresponds to a direction in the coordinate system, which is composed of geometric dimensions (X-, Y-direction) and a time-based dimension (Z-direction). Each measured value in the 3D-array corresponds to the measured amplitude at that point in millivolt [mV]. In addition to the 3D-array, 3 separate vectors are given that describe the respective geometric and time-based dimension. The X and Y values of the corresponding vectors are given in millimeters [mm]. The vector with the Z values contains the amplitudes, where time dimension is expressed in microseconds [µs].

**Fig. 1:**
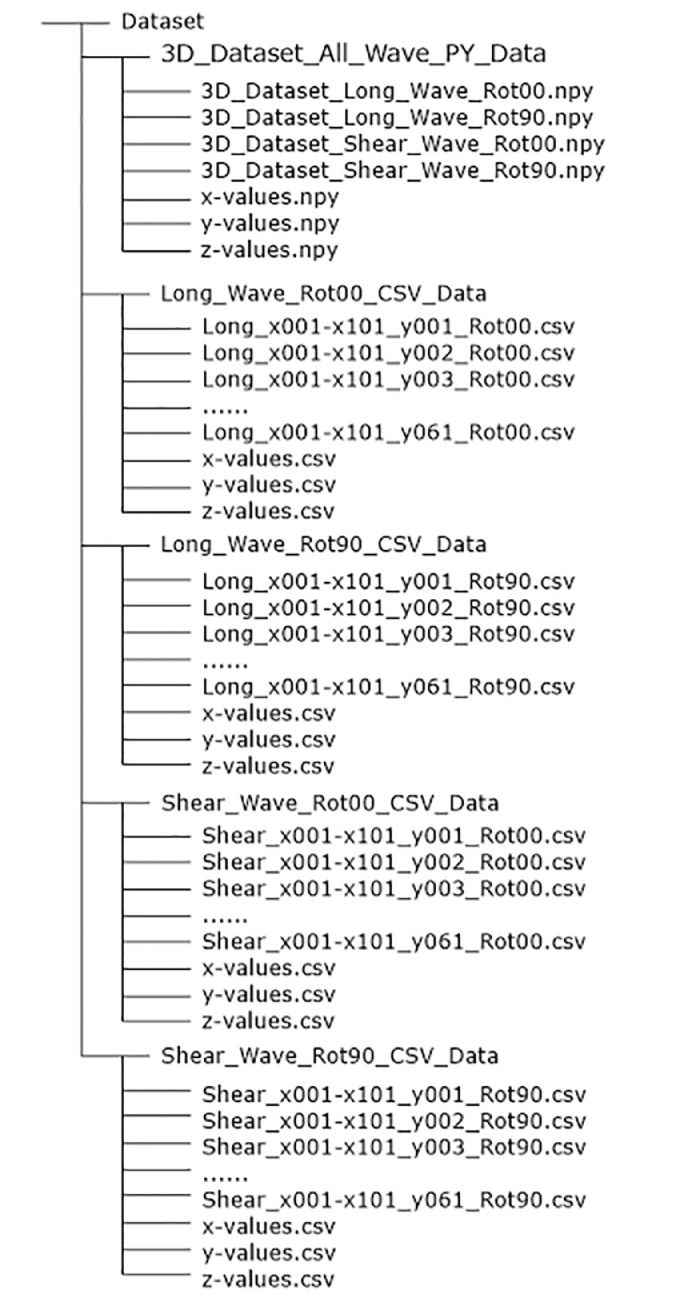
Tree of the data structure of the reprository [1].

**Fig. 2:**
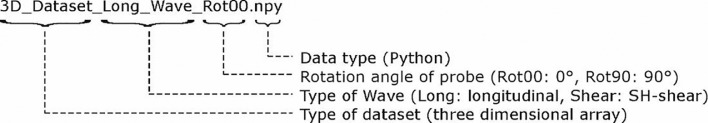
Example for the naming of a file in python format (*.npy).

The data structure of the folders, the subfolders and the files are shown in a tree structure in Fig. 1. The name of the datasets in Python format contains information about the type of dataset (3D), the type of the excited ultrasound wave and the orientation of the probe on the measurement surface. An exemplary Python file name is explained in Fig. 2.

**Fig. 3:**
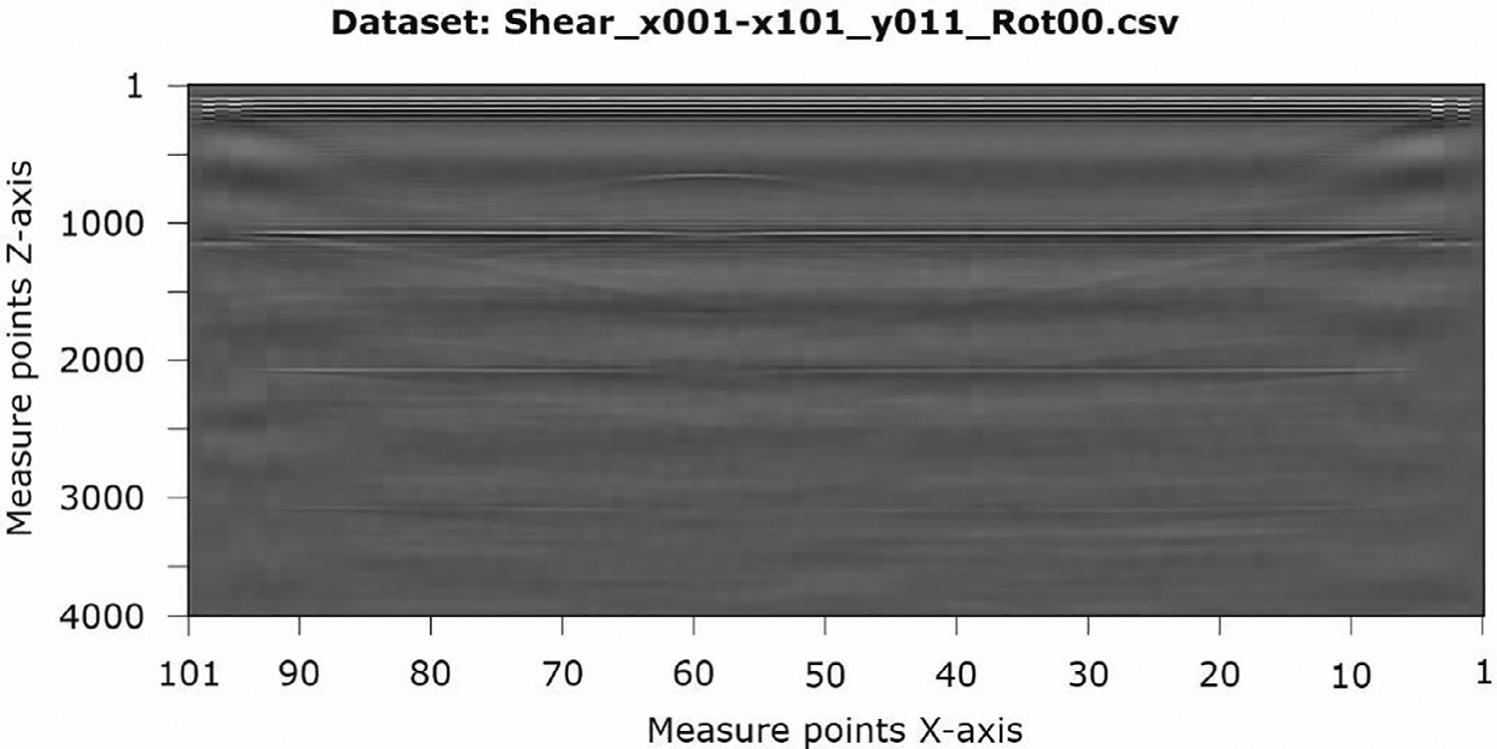
Example of the *.csv-dataset named “Shear_x001-x101_y011_Rot00.csv” imaged grey-scaled (B-scan).

The data, which are stored in *.csv format, have the shape of a 2D-array. For this, the measured values (amplitudes) in Z-direction are stored line by line for all measuring points in the X-direction. Thus, a dataset consists of 101 rows for the measurement points in X-direction with each 4000 amplitude values in Z-direction. Fig. 3 shows an example of the color-coded (gray-scaled) representation of a dataset (B-scan).

The filenames of these *.csv datasets contain information about the amount of the measuring points and the probe orientation.

**Fig. 4:**
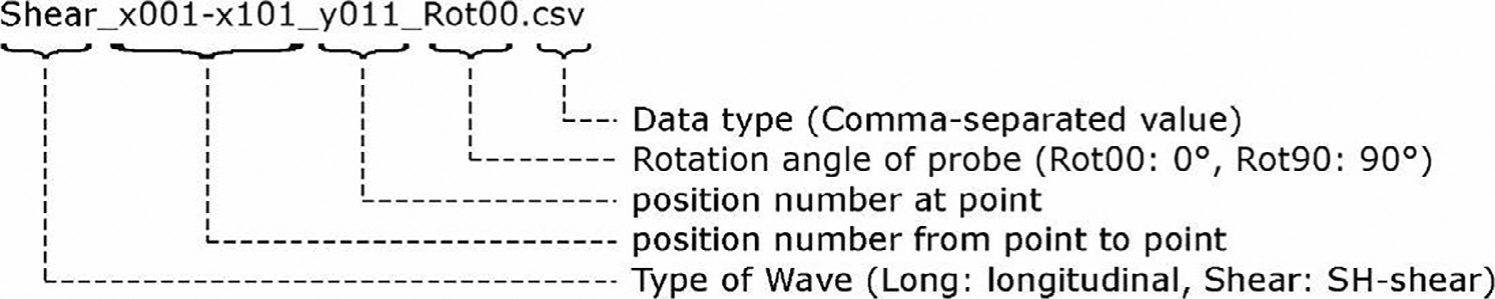
Example for the naming of a file in csv format (*.csv).

Fig. 4 explains the file naming scheme. In addition to the datasets of the individual measurement lines, 3 vectors are stored with the geometric and time dimension (Fig. 1, data tree). The X and Y values of the corresponding vectors are given in millimeters [mm]. The vector with the Z-values is given in microseconds [µs].

## 2 Experimental Design, Materials and Methods

### 2.1 Specimen

**Fig. 5:**
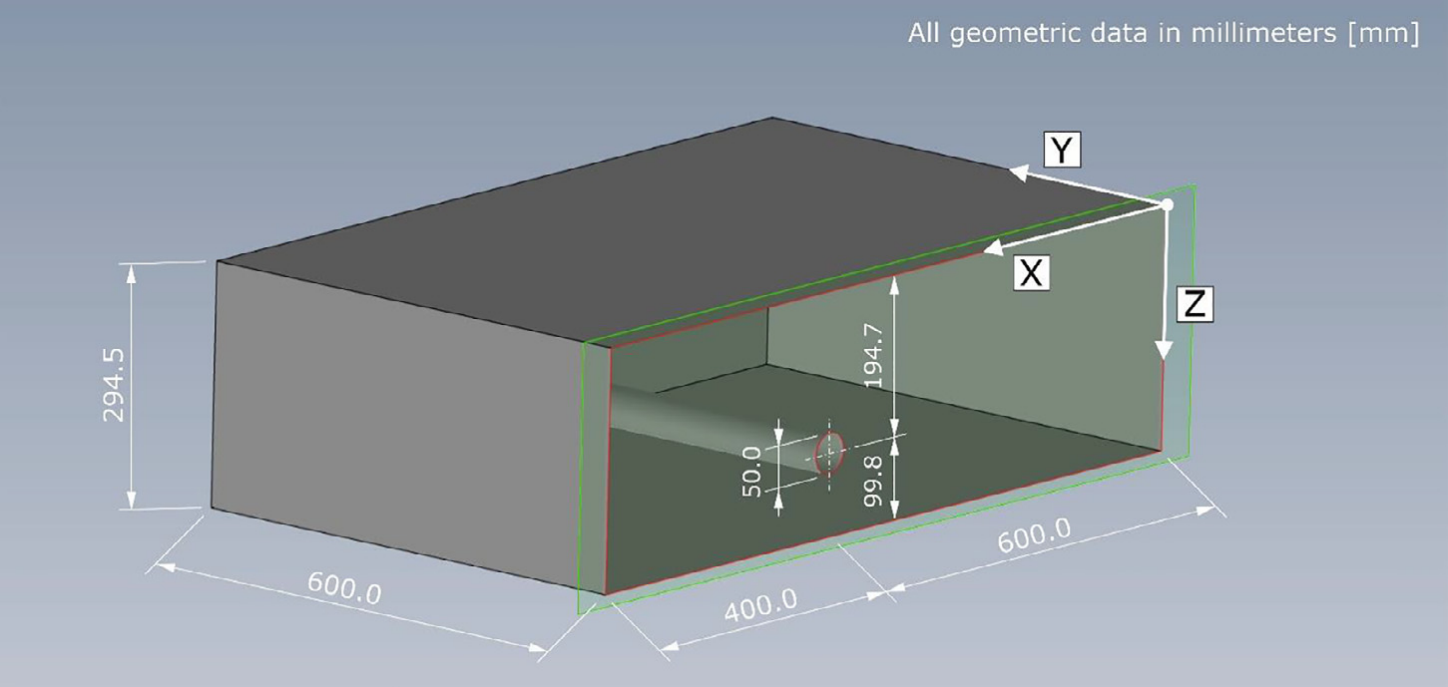
3D schematic representation of the geometric dimensions of the BAM reference specimen ”Pk218“ with borehole made of polyamide.

The specimen is composed of polyamide PA 6G and is shown schematically in Fig. 5. The material has a bulk density of *ρ* = 1.15 g/cm³ and a tensile modulus of elasticity of *E* = 3200 MPa [3].

All geometric descriptions refer to the right-handed coordinate system of the specimen with the axes X-Y-Z (Fig. 5) and are summarized in Table 1. The values for the geometrical dimensions, indicating the standard deviation additionally, are *l* = 1000 ± 0.03 mm (X-direction) in length, *b* = 600 ± 0.03 mm (Y-direction) in width and *h* = 294.5 ± 0.03 mm (Z-direction) in depth. The hole is parallel to the to the X-Y- and Y-Z-surface has a diameter of *D* = 50 ± 0.01 mm. The center of the hole is at a depth of *t* = 194.7 mm (Z-direction). The distance from the top of the hole to the surface at *z* = 0 mm is *t* = 169.7 ± 0.019 mm (Z-direction). The distance between the lower edge of the hole and the surface at *z* = 294.5 ± 0.03 mm is *t* = 74.8 ± 0.019 mm (Z-direction).

**Table 1:** Geometric dimensions of the test specimen ”Pk218“ with indication of the standard deviation.

Geometric dimension	X-direction	Y-direction	Z-direction
Length	1000.0 ± 0.03 mm	—	—
Width	—	600.0 ± 0.03 mm	—
Depth	—	—	294.5 ± 0.03 mm
Position bore hole^(*)^	600.0 ± 0.03 mm	—	194.7 ± 0.03 mm
^(*)^ Diameter 50.0 ± 0.01 mm			

### 2.2 Probe

**Fig. 6:**
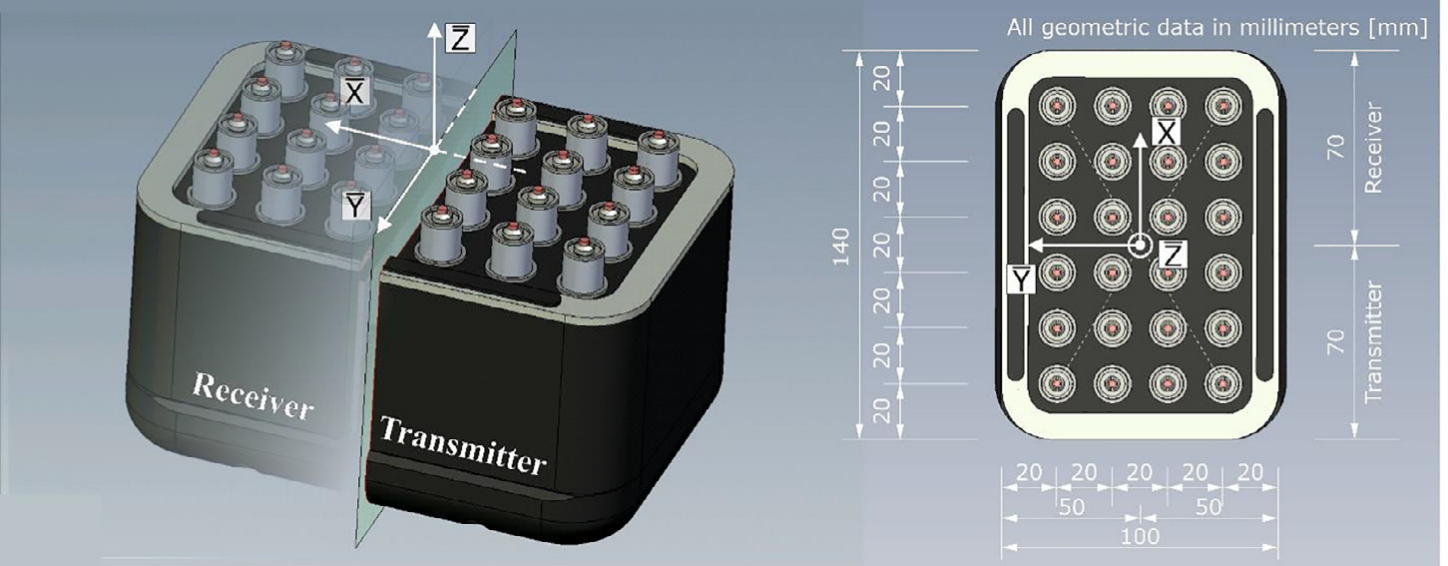
Schematic representation of the array in the view of the contact area and the associated coordinate system.

Fig. 6 shows two views of the ultrasonic probe. All geometric descriptions refer to the right-handed coordinate system of the probe with the axes X̅-Y̅-Z̅ and are summarized in Table 2.

**Table 2:** Geometric dimensions of probes.

Geometric dimension	X̅-direction	Y̅-direction
Probe - over all	*140.0 mm*	*100.0 mm*
Contact area – over all	*100.0 mm*	*60.0 mm*
Distance - single probes	*20.0 mm*	*20.0 mm*
Contact area - receiver	*40.0 mm*	*60.0 mm*
Contact area - transmitter	*40.0 mm*	*60.0 mm*
Ceramic tip single probe	∼1.7^(*)^ mm	∼1.7^(*)^ mm
		^(*)^ Diameter

The ultrasonic probe is an array consisting of 24 single probes that are spring-mounted to provide better contact to the specimen surface. 12 single probes are parallel-connected as transmitters and the other 12 single probes are parallel-connected as receivers. The single probes have a spacing of 20 mm related to the center of their ceramic tips. The area of the ceramic tip in contact with the specimen surface is approximately *D* = 1.7 mm in diameter. The probes operate based on the piezoelectric principle, do not require a coupling agent and can excite longitudinal (Probe M2503) and transverse (Probe M2502) waves [4]. Transverse waves are SH waves polarized in the Y̅-direction. The longitudinal wave is polarized in the Z̅-direction. The probe that excites a longitudinal wave has a nominal frequency of *f* = 100 kHz. The probe that excites a transverse wave has a nominal frequency of *f* = 55 kHz. Both probes have a bandwidth of about 60–70% [4]. The physical values are summarized in Table 3

**Table 3:** Physical values of probes [4].

Probe type	Wave form	Nom. frequency	Bandwidth	Polarization
M2503	longitudinal	*100.0 kHz*	*60–70%*	*Z̅-direction*
M2502	transversal	*55.0 kHz*	*60–70%*	*Y̅-direction*

**Fig. 7:**
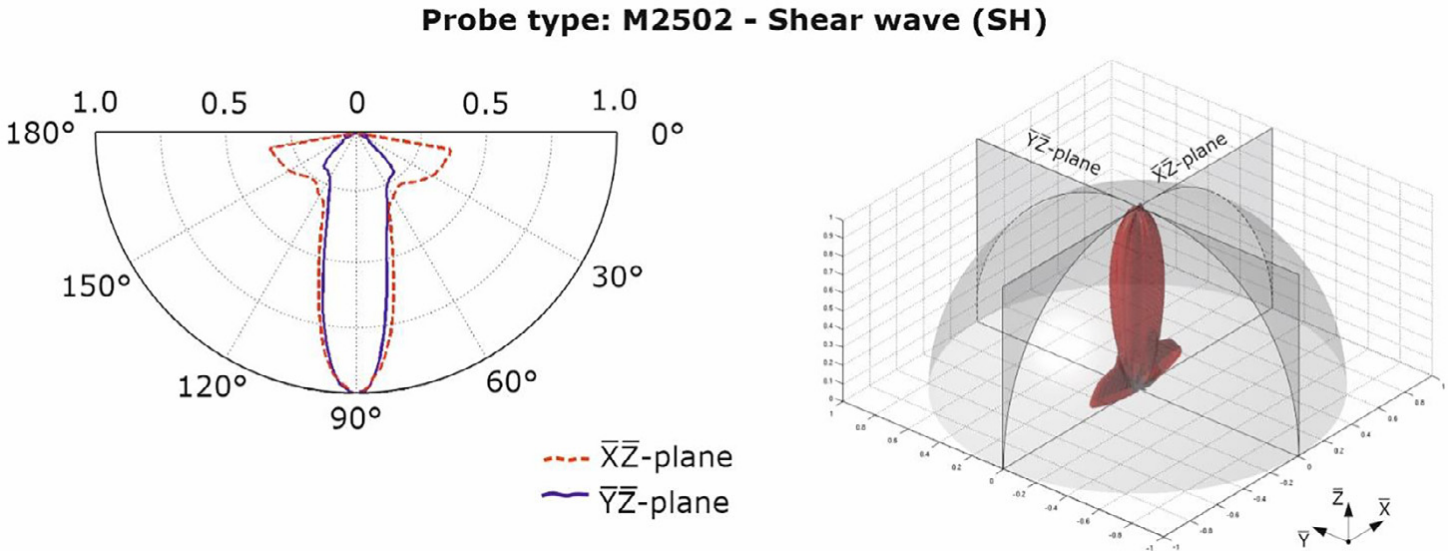
2D-directivity pattern of shear (transverse) wave probe M2502 [5].

**Fig. 8:**
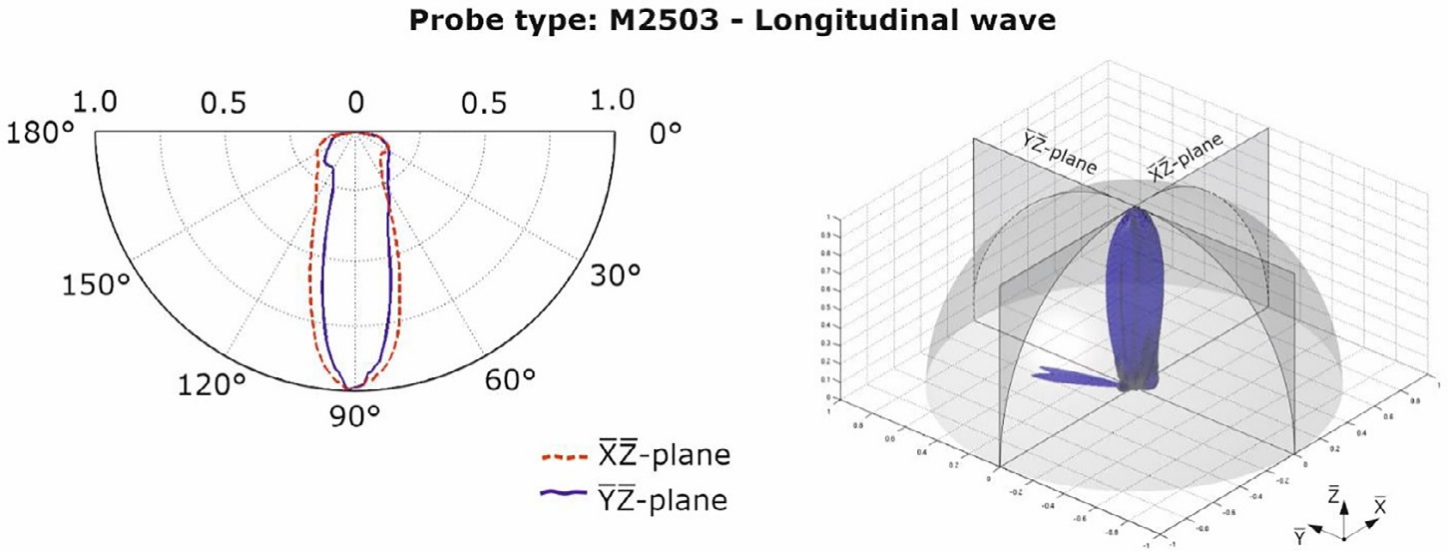
2D-directivity pattern of longitudinal wave probe M2503 [5].

The characteristic values of the sound fields depend on the design of the array. The operating principle of the single probe of an array can be taken from Shevaldykin et al. [4]. Fig. 7, Fig. 8 show the characteristic values (directivity pattern) for the longitudinal and transverse wave probes [5] measured in material polyamide PA 6G for two directions at a normalized amplitude value. The polar diagrams show the directional characteristics of the wave fields primarily excited by the probes. The directional characteristics of additionally excited wave types are not shown.

### 2.3 Automated Measurement Data Acquisition

**Fig. 9:**
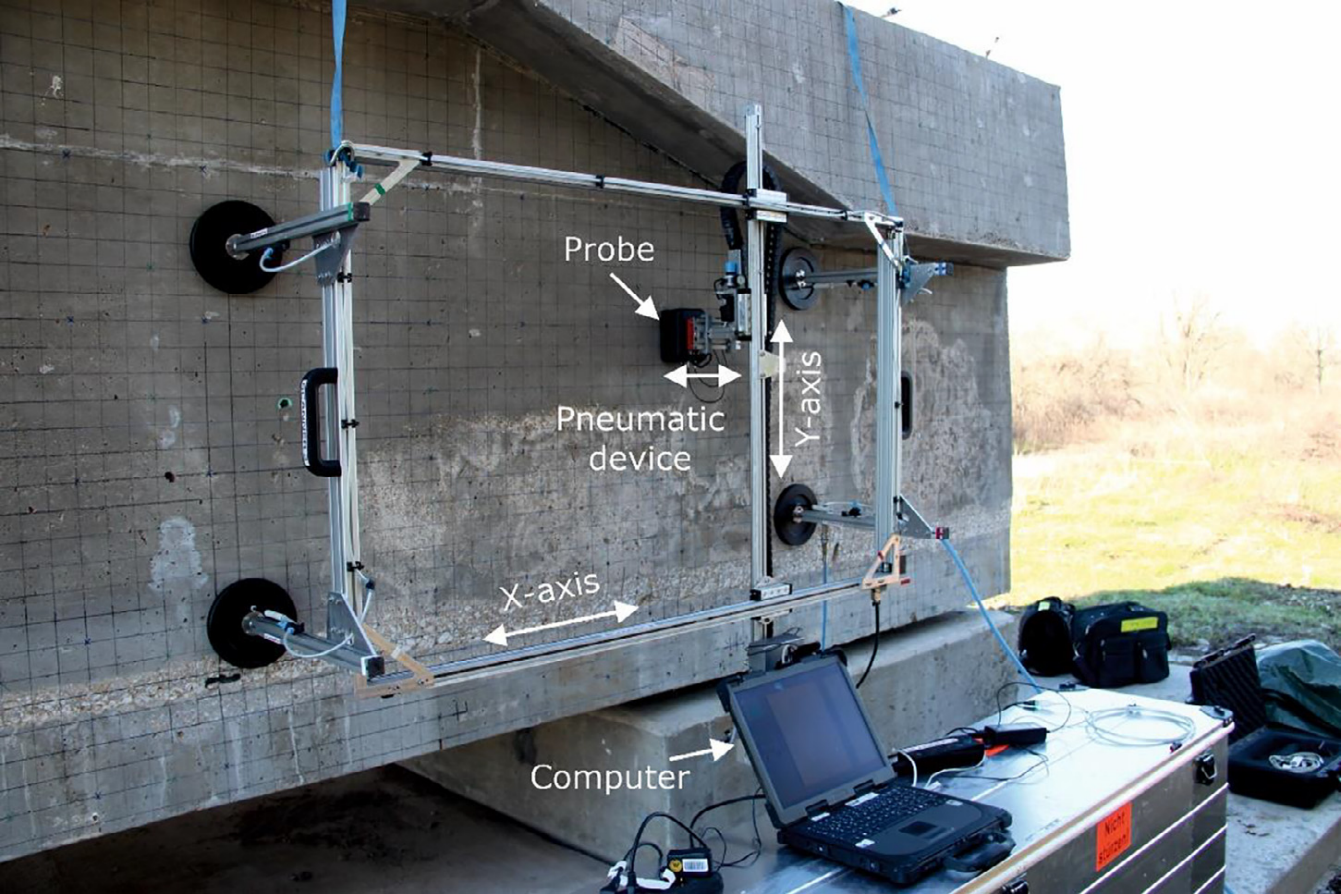
Automated scanner for ultrasound inspection mounted on a bridge girder mock-up specimen [6] which was used for the measurements on the ”Pk218“.

The measurements on the specimen were performed with a mobile automated scanner developed at BAM (Fig. 9). With the scanner, a measuring area of about 1 m² can be measured along two linear axes in a fully automated way. The array is pressed onto the surface by a pneumatic device with a constant contact pressure. The measuring grid can be variably adjusted*.*

The scanner is controlled and data is acquired via a LabVIEW software developed at BAM, which is installed on a commercial computer. The electrical pulses (rectangular pulse) with which the probes (M2502, M2503) are excited is generated by a pulse generator developed at BAM. The generated voltage of *V* = 100 V is transmitted to the probe by coaxial cable. The received pulse is sent to the data acquisition unit (USB-6361) and subsequently stored on the computer.

The scanner was used to scan the surface of the specimen with an equidistant measuring grid. The measuring point distance is *a* = 10 mm in the X- and Y-directions. The ultrasonic signals were recorded at each measuring position with 4000 sample values and a sampling rate of 2 MHz. The technical parameters of the automatic measurement are summarized in Table 4.

**Table 4:** Values of data aquisition.

Probe type	Measuring field dimension (X x Y)	Measuring point distance	Samples per measuring point	Sampling frequency
M2503	*1000* *mm x 600 mm*	*10 mm*	*4000*	*2 MHz*
M2502	*1000* *mm x 600 mm*	*10 mm*	*4000*	*2 MHz*

**Fig. 10:**
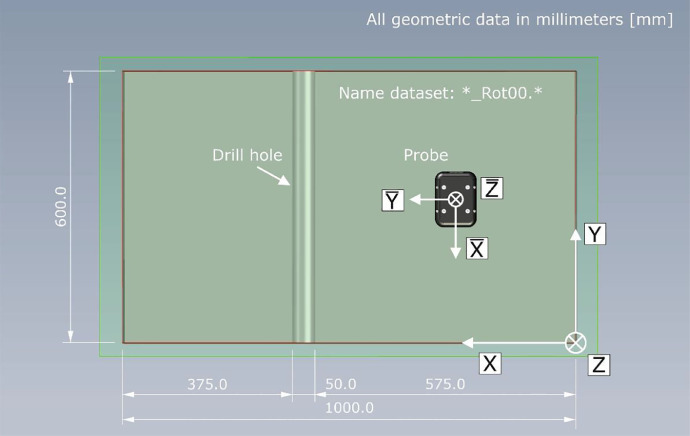
Top view on measurement area (red line) of specimen with geometric orientation of probe for dataset with name supplement *_Rot00.*.

For each excited wave type, the probe has been mounted in two perpendicular geometric orientations, so that a total of 4 datasets were recorded. Fig. 10 shows a schematic top view of the specimen with the geometric orientation of the probe for the data with the suffix ``*_Rot00.*''.

**Fig. 11:**
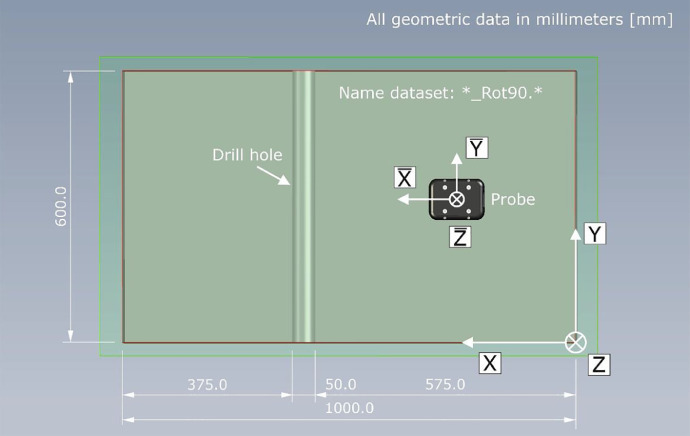
Top view on measurement area (red line) of specimen with geometric orientation of probe for dataset with name supplement *_Rot90.*.

Fig. 11 shows a schematic top view of the specimen with the geometric orientation of the probe for the data with the suffix "*_Rot90.*".

## Ethics Statements

These data are a primary data and does not include any human subjects, animal experiment, or social media platforms.

## CRediT authorship contribution statement

**Stefan Maack:** Conceptualization, Methodology, Investigation, Software, Formal analysis, Writing – original draft, Data curation. **Stefan Küttenbaum:** Formal analysis, Writing – review & editing. **Benjamin Bühling:** Formal analysis, Data curation, Writing – review & editing. **Ernst Niederleithinger:** Supervision, Writing – review & editing.

## Declaration of Competing Interest

The authors declare that they have no known competing financial interests or personal relationships that could have appeared to influence the work reported in this paper.

## Data Availability

Low-frequency ultrasound data (pulse-echo technique) with shear horizontal and longitudinal waves on a reference polyamide specimen “BAM-Pk218” (Original data) (Dataverse)
